# The Causes of Sudden Death

**Published:** 1897-07-03

**Authors:** 


### THE CAUSES OF SUDDEN DEATH.

There is a general feeling that man's natural end is to die in his bed, and most people look upon sudden death as something quite abnormal. It is well, then, to be reminded how many diseases tend to bring life to a sudden end; and in how many others, although sudden death may not be the ordinary termination, it is apt to occur as a result of complications which are not uncommon.

The whole subject has been fully treated by Professor Brouardel, and Professor Dixon Mann* has recently given a very complete account of the cases in which this accident may take place. A very large proportion of the sudden deaths which are met with are, of course, due to one or another form of heart disease. Next come apoplexy and other cognate diseases of the brain; and in regard to both heart and brain diseases, the frequent association of alcoholism is to be noted. Then there are the causes by which sudden asphyxia may be produced, a somewhat long list which includes many instances in which the obstruction to respiration arises not from any foreign body or morbid growth, but from mere spasm, such as occurs in whooping cough or laryngitis.

A curious cause of sudden death mentioned by Professor Mann, is hæmorrhage into the pancreas. It is most common in stout and alcoholic males over forty years of age, and up to the occurrence of the hæmorrhage the patients may appear in perfect health. People who suffer from enlargement of the spleen also are in constant danger of rupture of that organ, which may be caused by very trivial injuries, as, for example, by a mere pat with the palm of the hand.

Among the causes of rapid, although not generally actually sudden deaths, must be placed the rupture of a gastric or intestinal ulcer, or of an ectopic gestation, and the occurrence of coma from Bright's disease or diabetes. Cases of gastric ulcer may be absolutely devoid of symptoms until the moment when fatal rupture takes place. The same may be said of that very curious group of cases in which patients suffering from certain acute diseases go about until they drop—ambulant cases of pneumonia, typhoid fever, or effusion in the pleura. In pleuritic effusion the danger of sudden death on unaccustomed movement is very great indeed. Yet people go about with a full pleura, and, under such circumstances, the slightest hurry or the most trivial injury may cause immediate death. Pneumonia, again, may exist without preventing the patient from moving about, and such a condition, especially when occurring in old people and in combination with alcoholism, is a by no means uncommon cause of death in casual wards and police cells. A case is related of a man, aged 50, who called at a register office in search of employment, and fell down dead while the clerk was referring to the register, the whole of the upper lobe of the right lung being found on post-mortem examination to be in a state of grey hepatisation.

The liability to sudden death during and after attacks of diphtheria and influenza must always be remembered, and also the fact that, although in some cases a certain softness and flabbiness of the heart muscle may be discovered, in others nothing whatever may be found to account for death.

* Lancet, June, 1897.

The association of sudden death with a full stomach is curious and interesting, the actual cause of death generally being the regurgitation of vomited matters into the larynx and trachea. This is especially liable to happen to young children and to profoundly intoxicated persons.

The list of conditions in which sudden death may arise from rupture of cysts or abscesses either into the air passages or into the brain, or from accidents in the course of acute disease, is a long one, but in such cases a careful post-mortem examination will clear up all doubt.

Finally, mention must be made of the occurrence of sudden death from nervous shock, cases in which no injury has been inflicted during life, and no disease can be discovered after death. In the midst of excitement, or from fright, many apparently healthy people have died; but in regard to this the word excitement must be allowed to have a wider meaning than is usually given to it. Cases are on record in which patients have died during a mere vaginal examination, or during the passage of a catheter, and not a few have died on the operating table before they have been touched by the knife.

The relation of sudden death to coroners' inquests is a matter of some importance. Whenever a death occurs not only suddenly but unexpectedly, as in many of the cases just mentioned, some sort of inquiry will of course be held to ascertain the cause of the disaster. It is to be feared, however, that when sudden death overtakes peoples suffering from diseases which are recognised as liable to terminate suddenly, a very perfunctory sort of investigation is generally looked upon as sufficient, and it is impossible not to see to what dangers these people are exposed in consequence of the present condition of the law as to certification of death. No official inspection of the body takes place. If a medical man has been attending he is bound by law to give a certificate as to the cause of death, even although he may not feel sure that the patient is dead. Under such a law the sudden death of a person suffering from a disease likely to end suddenly is no bar to a certificate being given, and even if the medical man should refuse to give one, and an inquest should be held, we venture to say that there are not half a dozen coroners in England who would order a post-mortem examination in a case of sudden death occurring in the progress of a disease of which sudden death is a recognised termination. Thus it happens that those unfortunate persons who are known to be sufferers from such diseases—heart disease, for example—are practically outside the law so far as murder is concerned. We do not say that actual murder often happens, but we do say that the present condition of the law is a direct incentive to it. They are troublesome, wearisome patients to nurse; they involve an enormous amount of labour and self sacrifice upon their poor relatives, and they are in many cases so full of misery and so obviously sure to die that it is hard to keep down the thought that they would be better dead. In view of the trivial circumstances which appear sometimes to lead to murder, we cannot but see that the temptation to such a crime is comparatively great in cases such as we describe, where the life seems worthless, where its continuance is a trouble to all concerned, where the will is "all right," and the peevish patient is constantly threatening to change it, and where there is a practical certainty that no efficient investigation into the cause of death will ever be made. We cannot but think that this is a matter which demands reform.

